# Fungal Glycosidases in *Sporothrix* Species and *Candida albicans*

**DOI:** 10.3390/jof9090919

**Published:** 2023-09-12

**Authors:** Jorge A. Ortiz-Ramírez, Mayra Cuéllar-Cruz, Julio C. Villagómez-Castro, Everardo López-Romero

**Affiliations:** Departamento de Biología, División de Ciencias Naturales y Exactas, Universidad de Guanajuato, Guanajuato 36050, Mexico

**Keywords:** fungal glycosidases, *Sporothrix*, substrates, glycoproteins

## Abstract

Glycoside hydrolases (GHs) are enzymes that participate in many biological processes of fungi and other organisms by hydrolyzing glycosidic linkages in glycosides. They play fundamental roles in the degradation of carbohydrates and the assembly of glycoproteins and are important subjects of studies in molecular biology and biochemistry. Based on amino acid sequence similarities and 3-dimensional structures in the carbohydrate-active enzyme (CAZy), they have been classified in 171 families. Members of some of these families also exhibit the activity of trans-glycosydase or glycosyl transferase (GT), i.e., they create a new glycosidic bond in a substrate instead of breaking it. Fungal glycosidases are important for virulence by aiding tissue adhesion and colonization, nutrition, immune evasion, biofilm formation, toxin release, and antibiotic resistance. Here, we review fungal glycosidases with a particular emphasis on *Sporothrix* species and *C. albicans*, two well-recognized human pathogens. Covered issues include a brief account of *Sporothrix*, sporotrichosis, the different types of glycosidases, their substrates, and mechanism of action, recent advances in their identification and characterization, their potential biotechnological applications, and the limitations and challenges of their study given the rather poor available information.

## 1. Introduction

Glycosidases catalyze the hydrolysis of glycosidic bonds present in a variety of molecules such as carbohydrates, glycolipids, and glycoproteins [1]. By virtue of this property, most of these enzymes play a crucial role in numerous biological processes including the digestion of carbohydrates, the degradation of complex macromolecules, the regulation of cell signaling, and the modification of glycans during glycoprotein and glycoconjugate synthesis. There are different types of glycosidases that are named according to the glycosidic bond they break and the specific substrate they interact with. Some common glycosidases are α- and β-glycosidases, α- and β-mannosidases, β-galactosidase, β-xylosidase, β-fucosidase, and β-glucuronidase, among others, each exhibiting a characteristic mechanism of action [2]. Glycosidases have been classified in 171 families based on the similarities of amino acid sequence and 3D structures in the carbohydrate-active enzyme (http://www.cazy.org, accessed on 30 June 2023) database [2,3]. 

Knowledge of the mechanism of glycosidase action not only helps us grasp the biochemical intricacies at play but it also has practical implications in various fields, including biotechnology and drug development. At the core of substrate binding, active site residues orchestrate a complex interplay of non-covalent interactions—hydrogen bonding, van der Waals forces, and electrostatic attractions—establishing a substrate-specific environment akin to a lock-and-key mechanism. Substrate specificity is further refined by the positioning of active site residues to interact specifically with surrounding chemical groups, ensuring exclusive binding to the correct substrate and preventing nonspecific interactions. A deep, detailed insight into this process reveals that it involves several steps: (a) the enzyme–substrate complex is formed in an enzyme-specific site, (b) a water molecule approaches the glycosidic bond forming a glycosyl–enzyme–water ternary intermediate, and (c) an internal reorganization in the complex allows the nucleophile (water) access to the anomeric carbon of the substrate. This results in the break of the glycosidic bond and the release of an outgoing group while the remaining sugar bound to the nucleophile gives rise to a new molecule. The reaction may retain the sugar anomeric configuration as α → α or β → β or α → β or β → α, depending on the orientation of the nucleophilic attack and the relative position of the outgoing group in the substrate. It is worth noting that glycosidase itself plays a relevant role in providing a favorable environment and facilitating the correct orientation of the substrate for the hydrolytic reaction to occur in an efficient manner [1,4,5]. In fungi and most organisms, secreted or membrane-associated glycosidases play a fundamental role in the decomposition of organic matter and the use of products as sources of carbon and energy [4]. In pathogenic fungi, glycosidases are important for the invasion and colonization of animals and plant tissues, thus contributing to fungal virulence and evasion of the immunological defenses by altering the glycosidic moieties of immune system molecules [5,6].

## 2. *Sporothrix* and Sporotrichosis

### 2.1. General Aspects

**Figure 1:**
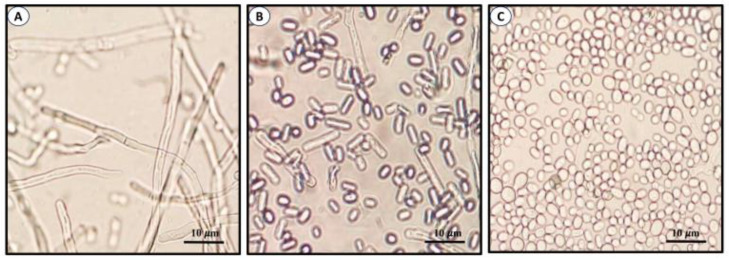
Morphological phases of *S. globosa*. Images show mycelia or filamentous cells (**A**), conidia (**B**), and yeast-like cells (**C**). Images produced in ELR laboratory.

*Sporothrix* belongs to the fungal class *Ascomycota* and the order *Ophiostomatales*. It is a genus formed by filamentous fungi found in soil, plants, and decaying organic matter and includes pathogenic species for both humans and animals as well as environmental members. In general, members of the environmental clade do not infect mammals, except for some members of the *S. pallida* and *S. stenoceras* complexes [7,8,9]. Species of the pathogenic clinical clade of the genus include *S. schenckii* sensu stricto, *S. brasiliensis*, *S. globosa,* and *S. luriei*, which are responsible for the mycosis known as sporotrichosis of humans and animals, such as cats and dogs [10,11,12,13]. These species exhibit variations in virulence, transmission, and geographical distribution. *Sporothrix* is a true dimorphic fungus whose morphology depends on temperature, pH, and other factors, such as cyclic nucleotides [13,14]. At 25–28 °C and an acidic pH, it develops as mycelium, which is considered as the saprophytic phase whereas at higher temperatures (32–37 °C) and a pH of 7.0–7.5, it grows as yeast-like cells, which is the morphotype frequently isolated from infected tissues [13,14,15]. Hyphae form either primary or sympodial and secondary or sessile conidia [16,17,18,19,20]. Figure 1 shows the morphological stages of *S. globosa*, which are similar to other members of the genus.

Sporotrichosis is a cosmopolitan mycosis of humans and animals caused by members of the pathogenic clade of *Sporothrix* and is common mainly in intertropical areas. The pathogen is transmitted mainly in two ways: (a) the classical route, where soil- or decaying organic matter-borne fungal propagules (sapronosis) are inoculated through trauma and lacerations, and (b) an alternative route, in which the pathogen is inoculated via scratches or bites by infected animals. This route explains the horizontal transmission and zoonosis produced mainly by domestic cats [11,12,16]. On the other hand, infection by *S. brasiliensis*, an emerging fungal pathogen, can occur exclusively via bite, scratch, spore inhalation, direct contact with secretion, etc. [21]. 

### 2.2. Clinical Manifestations of Sporotrichosis

**Figure 2:**
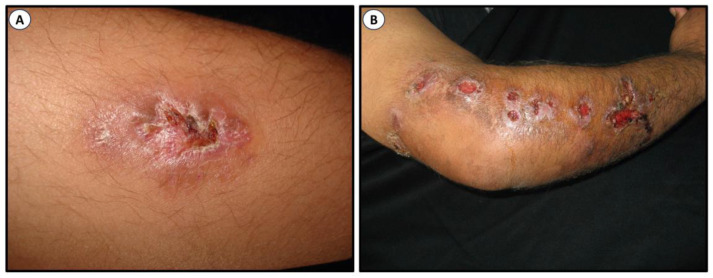
Clinical lesions of sporotrichosis. Fixed cutaneous (**A**) and classical lymphangitic or subcutaneous (**B**). Images were kindly provided by Dr. Alexandro Bonifaz, Mexico.

Sporotrichosis is a benign mycosis of humans. In general, it is limited to the epidermis where it forms lesions, the subcutaneous tissue, and the adjacent lymphatic vessels with occasional dissemination to bone tissue and internal organs. The severity and characteristics of the injuries depend on factors such as the depth of the lesion, the inoculum size, the inoculated morphology, virulence, comorbidities, and the immunological state of the host [22,23,24,25,26,27]. Sporotrichosis has been classified into four clinical categories: (a) fixed cutaneous, (b) lymphocutaneous, (c) multifocal, and (d) extracutaneous (systemic or visceral). It has a bad prognosis as it is associated with immunosuppression [24,25,26,28,29,30,31]. Figure 2 illustrates two of the most common clinical lesions of sporotrichosis.

### 2.3. The Cell Wall

In most fungi, the cell wall (CW) plays a fundamental role in the pathogenicity and virulence of *Sporothrix* and other fungi, as it establishes the first contact with the host. It is formed by two layers: a relatively conserved internal layer containing β1,3- and β1,6-glucan, which is alkali-insoluble and linked to chitin by β1,4-linkages, forming a very dynamic exoskeleton that protects the cell from internal (cell membrane and cytoplasmic turgor) and external factors. The external layer is more heterogeneous and adaptable to physiological conditions. Apart from these components, the CW also contains galactomannans, glycoproteins, glycolipids, and melanin [32]. The fungal CW plays a vital role in host–pathogen interaction at most stages of infection, aiding in keeping cell integrity [33]. Various pathogen-associated molecular patterns (PAMPs) are present on the cell surface, which could be recognized by the host immune system’s pattern recognition receptors (PRRs), or they can function as virulence factors, contributing to the infection process and influencing the organism’s pathogenicity [34,35,36]. The proteins deriving from the *Sporothrix* CW remain insufficiently defined; however, multiple accounts exist regarding specific adhesins that attach to host extracellular matrix (ECM) proteins, including fibronectin, laminin, and type II collagen [37,38,39,40]. Pathogen adhesion to host cells plays a pivotal role in proper colonization and subsequent spread [40]. 

The β-glucan layer is joined to two classes of proteins: those containing internal repetitions (PIR) and those dependent on glycosil-phosphatidylinositol (GPI) that couple covalently to polysaccharides. Functions such as cell adhesion, masking, and immune blocking have been attributed to these extensions [41,42,43,44,45,46,47,48]. In *S. schenckii* and *S. brasiliensis*, the CW structure includes β-glucan microfibrils with β1,3, β1,4, and β1,6 linkages, as well as chitin and a peptidorhamnomannan (PRM), in addition to other homogeneous and heterogeneous polymers and manoproteins. Polysaccharides constitute approximately 80% of the dry weight of the CW, while glycoproteins constitute 20%. In some *Sporothrix* species, there may be variations, as some lack α-glucan. The absence of α-glucans in the CW of *Sporothrix* could have diverse consequences on the pathogen physiology, affecting its structural integrity, ability to withstand stress, interaction with the host immune system, colonization efficiency, and other aspects such as energy storage, virulence, and cell signaling [32,41,44,49,50,51,52,53,54,55,56]. Recently, the CW composition was analyzed in conidia, germlings, and yeast-like cells of *S. globosa* and compared with *S. schenckii* and *S. brasiliensis*. It was found that both conidia and yeast-like cells of *S. globosa* had a higher amount of chitin in their CWs, and all morphologies had more exposed β1,3-glucan on their surface compared to *S. schenckii* and *S. brasiliensis* [57]. The composition and structure of the fungal cell wall are influenced not only by environmental conditions but also by the cell cycle, changes in growth form, and other processes [58,59,60,61]. 

### 2.4. Pathogenicity and Virulence

Virulence factors have been recently reviewed in the hyphae, conidia, and yeast-like cells of *S. schenckii* [62]. Accordingly, while chitin and β1,3-glucans are associated with the virulence of conidia and hyphae, other components are responsible for the virulence of yeast-like cells. These include the antigenic PRM, CW proteins, melanin, secreted and intracellular proteases, extracellular vesicles, and some lipids. Many of the cell surface components are glycoproteins that allow *Sporothrix* to interact with the host and evade the immune response, thus allowing the pathogen to survive. Some of these virulence factors are discussed below.

Adhesion of the pathogen to target cells is a required condition for fungal infection to occur. Thus, blocking this primary step represents a potential target to prevent it. Some of the CW proteins, generically known as adhesins, bind to host extracellular matrix (ECM) proteins such as laminin, elastin, fibrinogen, fibrinonectin, and others. One of the best-known adhesins is Gp70, which is one of the major antigenic components of the CW and an important immunogenic factor against *Sporothrix*. Gp70 adhesin was purified, shown to contain 5.7% *N*-linked oligosaccharides, and shown to be involved in the adhesion of yeast cells to mouse tail dermis [63]. Its presence was further demonstrated in *S. schenckii*, *S. brasiliensis*, and *S. globosa* [64,65]. Later, recombinant Gp70 was expressed in *E. coli,* and its gene was predicted to encode for a 43 kDa protein. It was immunogenic and contrary to previous findings and contained a much higher amount of oligosaccharide [66]. Thus, Gp70 seems to play the dual role of an adhesin and antigen. 

A major antigenic component of *S. schenckii* CW is PRM, a complex glycoconjugate that was purified from the yeast morphotype and shown to contain 57% mannose, 33.5% rhamnose, 14.2% proteins, and 1% galactose [53,67]. Whereas the sugar composition of RPM has been fully characterized, only a few of the constituent 325 proteins have been identified. Of these, recombinant GroEL/Hsp 60 and the uncharacterized protein Pap1 showed adhesion to ECM proteins and were more abundant in the yeast morphotype during interaction with HeLa cells [28]. 

A very important virulence factor is dimorphism, which is defined as the ability of certain fungi to switch between unicellular yeast and multicellular filamentous growth, depending on some environmental conditions. Seemingly, the change in the saprophyte to the parasitic form in pathogenic fungi obeys the need of the organism to adapt, grow, and disseminate in internal organs. The fungus enters the host in the form of conidia or short hyphae which, after some time, transforms into yeast-like cells. It is not clear whether this shift occurs extracellularly and then yeast cells are phagocyted or if conidia and hyphae are first internalized and the dimorphic transition occurs inside the cell. Some results suggest that the conversion of conidia to yeast and/or mycelium can occur inside macrophages [68]. 

In studies to investigate the compensatory cell responses to damage of the CW by perturbing agents, it was observed that 15 μM Congo red inhibits conidia germination of *S. schenckii* under conditions set for yeast development but not for mycelial growth, even at a 10-fold higher concentration. When the dye was added to yeast cells pre-grown in its absence, cells rapidly differentiated into mycelial cells, suggesting that this shift may be a strategy to evade the noxious effect of the dye [69]. Further studies confirmed the same behavior in *S. globosa* and showed that hypha returned to yeast-like cells as soon as the dye disappeared. Cell compensatory responses also included significant variations in the activity of glucosamine-6-phosphate synthase, a critical regulatory enzyme of UDP-GlcNAc levels [70,71]. 

Melanin is a factor of virulence present in the CW of *Sporothrix* and many other pathogenic fungi. For its importance in pathogenesis, this pigment has been studied from different aspects including its role in cutaneous sporotrichosis [72], synthesis and assembly in fungi [73], structure [74], biosynthesis in pathogenic species of *Sporothrix* [75], its relationship with the mammalian immune system [76], its synthesis pathway in fungi as a source for fungal toxins [77], and its role as a factor of virulence in *S. schenckii* [62]. Melanins are structurally complex dark pigment polymers present in all biological systems [78,79] and in fungi, they are synthesized by two pathways, either from 1,8-dihydronaphthalene (DHN) or L-3,4-dihydrophenylalanine (L-DOPA) [76]. The synthesis of pyomelanin in fungi involves oxidizing and polymerizing aromatic amino acids, like tyrosine, forming a three-dimensional melanin network. Pyomelanin provides protective and adaptive properties, helping the fungus resist environmental factors and antifungal agents. Precise synthesis details can vary by fungal species and environmental conditions [80]. In one study, nonpigmented *S. schenckii* was phagocyted more readily by human monocytes and murine macrophages than its melanized counterpart. It was also observed that the pigment protects the fungus against radiation [81]. In the same line, several characteristics of sporotrichosis induced in rats with wild type (MEL^+^) and mutant (MEL^-^) strains of *S. schenckii* were compared. Among other observed differences, the pigmented strain showed greater tissue invasion giving rise to multifocal granulomas, while the mutant cells restricted the fungus to the core of the granulomas [72]. Melanin also protects fungal cells from reactive oxygen species and nitric oxide released during phagocytosis [62]. Moreover, it has been observed in some fungi that melanin interferes with the immune system as it reduces the effectiveness of phagocytes, binds effector molecules and antifungals, and alters complement and antibody responses [76]. 

**Figure 3:**
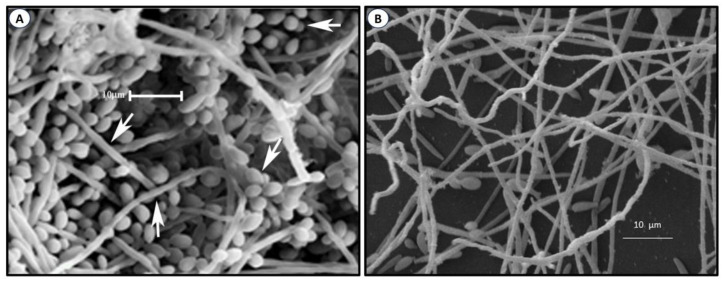
Biofilm formed by *C. albicans* (**A**). Adapted with permission from [89], under an open access Creative Common License Deed (CC BY 3.0). Copyright 2015 Hindawi. *S. schenckii* (**B**) was produced in the JCVC laboratory.

The most important factor of the virulence of *Sporothix* and many other organisms is the formation of biofilms. These are highly organized structures formed by sessile cells and a variable amount of extracellular polymeric substances (EPS), which function as an impermeable protecting coat that limits the diffusion of chemical substances, leading to recurring infections and resistance to antifungals [82,83,84]. Since the discovery of biofilms, it has been learned that about 90% of microorganisms possess this ability. The formation of biofilms is a complex process that occurs in four phases, namely adhesion, aggregation, maturation, and disaggregation. These colonies of sessile cells can form either on biotic or abiotic surfaces [85,86,87,88], such as medical devices, including catheters, prothesis, pacemakers, and others. A number of properties of biofilms have been studied in several pathogens, particularly *Candida albicans* and other pathogenic species of the genus [87,88,89,90,91] A biofilm formed by *C. albicans* is shown in Figure 3A. Recently, a review of materials used to prevent adhesion, growth, and biofilm formation of *Candida* species [92] was published. Information on biofilm formation by *Sporothrix* is rather limited. An image of a *Sporothrix schenckii* biofilm is depicted in Figure 3B. In one study, the ability of *Sporothrix* spp. to form biofilms in vitro was investigated, as well as the growth, morphology, and sensitivity of sessile cells to antifungals. Results corroborated previous findings observed in other organisms, i.e., that *S. schenckii* formed well-structured biofilms and the growth of sessile cells was less sensitive to antifungals than planktonic cells [93,94]. Later, it was demonstrated that biofilms formed in vitro by both mycelia and the yeast-like cells of the *S. schenckii* complex are inhibited by potassium iodide and miltefosine, an antiparasitary drug effective against helminthiasis [95]. Chitosan exhibits antimicrobial activity against many organisms, a function that largely depends on its molecular weight (MW) and deacetylation degree (DD) [96]. In one experiment, low, medium, and high MW chitosans with DD of 75–85% were tested in ten isolates of *S. brasiliensis* in the filamentous phase. Their effect was measured on planktonic cells, initial adhesion for biofilm formation, and mature biofilms. Low MW chitosan was more inhibitory of both planktonic cells and biofilm formation than the other chitosans, suggesting that low MW chitosan can penetrate and interact with the biofilm more easily than the high MW form, leading to the destruction of the biofilm structure more efficiently [97]. 

## 3. Glycosidases

### 3.1. General Mechanism of Action

**Figure 4:**
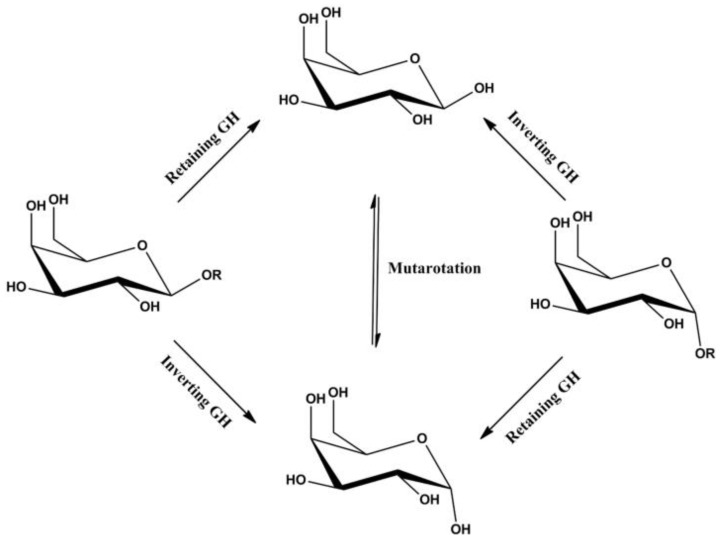
Catalytic mechanism of retaining and inverting glycosidases. Reprinted with permissionfrom [5], under an open access Creative Commons Attibution 3.0. Copyright 2012 IntechOpen.

A brief account of the general mechanism of reaction of glycosidases is given at the beginning of this review. Here, we go deeper into the fine-tuning of this catalytic process. As mentioned earlier, the hydrolysis of the glycosidic bond can lead to two stereochemical results: retention or inversion of the anomeric configuration, which implies the operation of at least two distinct mechanisms, as illustrated in Figure 4. However, it has been observed that all glycosidases belonging to the same sequence-related family give rise to the same result. In those enzymes that invert configuration, the two carboxyl groups act as general acids and base catalysts suitably positioned at an average distance of 10.5 Å to allow the substrate and a water molecule to bind between them. A reaction occurs by a mechanism involving an oxocarbenio ion-like transition state. In glycosidases that retain configuration, the two carboxyl groups are separated by 5.5 Å, which is consistent with a double displacement mechanism involving a covalent glycosyl enzyme intermediate. In the first step, one of the carboxyl groups acts as a general acid catalyst, protonating the glycosidic oxygen simultaneously with bond cleavage. The other acts as a nucleophile, forming a covalent glycosyl–enzyme intermediate. In the second step, the side-chain carboxylate deprotonates the incoming water molecule, which attacks at the anomeric center and displaces the sugar [4].

### 3.2. Glycosidases and Glycoprotein Synthesis

Protein glycosylation stands as a remarkably preserved post-translational alteration observed in both prokaryotic and eukaryotic cells. This process entails the attachment of oligosaccharides and glycolipids to the peptide backbone. The impact of glycans extends to a range of biological phenomena, including protein folding, precise transport, cellular positioning, and the facilitation of host–pathogen interactions, among other vital functions [35]. There are two types of glycosylation: *N*-linked and *O*-linked. The attachment of *N*-glycans starts in the endoplasmic reticulum (ER), where the core glycan is joined by means of two moieties of *N*-acetylglucosamine (GlcNAc) to a specific asparagine residue of the nascent protein bearing the NXS/T sequence, or *sequon*, where X can be any amino acid except proline. *N*-glycans are trimmed by specific glycosidases and further modified as they transit through the ER and the Golgi complex. *O*-glycosylation occurs in the Golgi apparatus where the core *O*-glycans are attached through *N*-acetylgalactosamine (GalNAc) to the side chain of serine or threonine residues of the protein [98]. 

Early studies on the role of glycosidases in glycoprotein synthesis in mammalian cells [99] and on the structure and function of mannosidases in *Saccharomyces cerevisiae* and mammalian cells in glycoprotein synthesis and quality control [100] paved the way for further studies of these enzymes in various organisms. Many of these have considered *S. cerevisiae* as a reference for these studies. The generation of mutants, the analysis of phenotypes, and the effect on the physiology of mutant cells have been common approaches to investigating the role of glycosidases in glycoprotein synthesis.

The most common enzyme to remove *N*-glycans from a glycoprotein is peptide-*N*-glycosidase F (PNGase F). This amidase breaks the link between the innermost GlcNAc residue and the asparagine of the peptide, releasing the *N*-glycan and converting asparagine into aspartic acid. Released *N*-glycan can then be analyzed by LC-FLD and/or LC-MS, CE-LIF, and other techniques. Another enzyme, known as endo-β-*N*-acetylglucosaminidase (ENGase), breaks between the two GlcNAc residues, leaving one GlcNAc residue attached to the protein. The specificity of these enzymes is limited to the type of *N*-glycans they recognize. No endo-β-*N*-acetylglucosaminidase can break *O*-glycans, making the study of these glycoconjugates more difficult. On the other hand, there are two *O*-glycosidases: one from *Streptococcus pneumoniae* and the other from *Enterococcus faecalis*. The first releases galactose β1,3-linked to GalNAc, while *E. faecalis*, with a broader specificity, releases a GlcNAc-β1,3-GalNAc disaccharide [101,102].

### 3.3. Glycosidases in Sporothrix Species

Information on glycosidases involved in protein glycosylation in the pathogens referred to in this review has been generated in our laboratory and those of others. Regarding α-mannosidases, two types of these glycosidases belonging to family GH47 are involved in the trimming of *N*-glycans: those that trim only one mannose from Man_9_GlcNAc_2_ (M_9_), forming Man_8_GlcNAc_2_ isomer B (M_8_B) [103,104] in the ER, and those that operate in the Golgi complex in mammalian and fungal cells that remove three α1,2-linked mannoses from Man_8_GlcNAc_2_, forming Man_5_GlcNAc_2_, a primer required for the synthesis of complex and hybrid *N*-glycans [103,105]. In *S. schenckii*, a membrane-bound α-mannosidase of 75 kDa was solubilized from the ER, purified to homogeneity, and biochemically characterized. The enzyme was shown to convert M_9_ into M_8_ isomer B and was preferentially inhibited by deoxymannojirimycin. To our knowledge, this was the first report on the isolation and biochemical analysis of an enzyme involved in the *N*-linked protein glycosylation in *S. schenckii* [106]. 

In contrast to other filamentous fungi, *S. schenckii* lacks a Golgi α-mannosidase, suggesting that the processing of *N*-glycans by these enzymes is similar to lower eukaryotes, such as *C. albicans* and *S. cerevisiae,* which also lack these glycosidases in the Golgi and form only high-mannose *N*-glycans [106,107].

Early steps of *N*-linked glycoprotein synthesis involve the transfer of the dolichol-PP-anchored Man_9_GlcNAc_2_Glc_3_ (Glc3Man9) oligosaccharide to specific asparagine residues of nascent proteins in the ER. Shortly after the transfer, the terminal α1,2-linked and the two more internal α1,3-linked glucose residues are specifically removed by the sequential action of ER-resident α-glucosidases I and II, respectively [99,107]. These steps are similar in yeast, plant, and mammalian cells [108]. Further, α1,2-mannosidase selectively eliminates a specific mannose residue, generating the Man_8_GlcNAc_2_ isomer B (M_8_B) oligosaccharide [99], which either exits the ER for further modification in the Golgi complex or is targeted for degradation if is not properly folded in a process known as ERAD (ER-associated degradation) that involves α-glucosidases I and II [109].

Genetic and molecular biology approaches to the study of glycosidases involved in protein glycosylation were not possible until the genomes *S. schenckii* and *S. brasiliensis* were sequenced and a database was available. This achievement allowed us to use *S. schenckii* sensu stricto to track members of families GH 47 and 63 that gather glycosidases that are potentially involved in glycoprotein processing [110]. These authors detected eight homolog genes, seven belonging to family 47 and one to family 63. To determine the functional characterization of the genes, they used null mutants of *C. albicans* and investigated the ability of genes to restore the wild type phenotype. To that purpose, members of family 47 and one of family 63 were individually expressed in mutants lacking *MNS1* and *CWH41*, respectively. They found that genes Ss*MNS1* and Ss*CWH41* were the orthologs of Ca*MNS1* and Ca*CWH41*, respectively. They also observed that other members of family 47 encode Golgi mannosidases and RE degradation-enhancing a-mannosidase-like proteins (EDEMs) [109]. A benefit derived from the identification of the genes hosted by these families is their potential role as targets for antifungals against infectious diseases.

As mentioned above, α-glucosidases I and II play a fundamental role in *N*-glycoprotein biosynthesis, in particular α-glucosidase II, which acts after α-glucosidase I by trimming the two α1,3-linked glucose residues. This generates a deglycosylated intermediate, which is further processed by ER α1-2-mannosidase and fully elongated by Golgi mannosyltransferases [33,111]. α-Glucosidase II is also involved in the ER quality control system for misfolded proteins, which are further degraded in the proteasome [109,112]. In most eukaryotes, α-glucosidase II is a heterodimer consisting of α and β subunits, which correspond to the catalytic site and the ER retention signal, respectively [113].

A distribution analysis of α-glucosidase activity in the yeast morphotype of *S. schenckii* was carried out by a fluorometric method using 4-methylumbelliferyl-α-D-glucopyranoside (MUαGlc) as a substrate [114]. The results revealed that 38% and 50% of total enzyme activity were present in a soluble and a mixed membrane fraction (MMF), respectively. The MMF-bound enzyme was solubilized with the nonionic detergent Lubrol WX and purified to homogeneity to a dimeric protein consisting of 75.4 and 152.7 kDa subunits. Analysis of hydrolysis of α-linked glucose disaccharides such as nigerose (1,3), kojibiose (1,2), trehalose (1,1), and isomaltose (1,6) revealed the highest preference for nigerose over the other substrates, suggesting that it was an α-glucosidase II. Further evidence was obtained after using selective inhibitors of processing α-glucosidases, namely 1-deoxynojirimycin, castanospermine, and australine. Accordingly, 1-deoxynojirimycin, a more specific inhibitor of α-glucosidase II than I, was a stronger inhibitor of the hydrolysis of both MUαGlc and nigerose than the other inhibitors. Taken together, these properties are consistent with a type II-like α-glucosidase that was probably involved in *N*-glycan processing. To our knowledge, this was the first report of this activity in a truly dimorphic fungus [114]. 

In a further study, degenerate primers and inverse PCR approaches were used to isolate the open reading frame of *ROT2*, the encoding gene for the α subunit of ER α-glucosidase II in *S. schenckii* [114]. This approach revealed that *ROT2* complemented a *S. cerevisiae rot2∆* mutant and when expressed in *C. albicans rot2∆* mutant, it partially increased α-glucosidase activity but failed to restore the *N*-glycosylation defect. This was the first report on the isolation of a gene involved in glycoprotein assembly in *S. schenckii* [115]. Recently, López-Ramírez et al. [116] were able to silence *ROT2* and investigate how the lack of the catalytic subunit of RE α-glucosidase II affects the CW, host interaction, and virulence of *S. schenckii* as tested in *Galleria mellonella* larvae. They obtained three strains with intermediate (47.9 to 59.3%) and three with high (98.9 to 99.6%) *ROT* silencing. Gene silencing resulted in the accumulation of the Glc_2_Man_9_GlcNAc_2_ glycan core and a decrease in *N*-linked glycans in the CW; yet high-silenced strains showed a compensatory increase in CW *O*-linked glycans. *ROT* silencing also reduced cytokine production, macrophage phagocytosis, and virulence. The silencing of *ROT*, however, did not affect *S. schenckii* growth and morphology [116]. 

### 3.4. α-Glycosidases in C. albicans 

Back in 1988, the soluble form of a specific α-mannosidase (Fraction II), a glycosidase that removes mannose from Man_9_GlcNAc to form a single isomer of Man_8_GlcNAc, was purified and characterized from *S. cerevisiae* [117]. Using this report as a starting point, Vázquez-Reyna et al. [118] later described that about 80% of α-mannosidase activity in the yeast cells of *C. albicans* was in a soluble form and demonstrated that no part of this enzyme resulted from the proteolytic release of a particulate cell component. These authors used 4-methylumbelliferyl-α-D-mannopyranoside and *p*-nitrophenyl-α-D-mannopyranoside as substrates and observed that the hydrolysis of the fluorogenic substrate was strongly inhibited by 1-deoxymannojirimycin (67%) and swainsonine (83%), whereas the hydrolysis of the colorimetric substrate was reduced to a much lower extent (10–15%) by both inhibitors. A molecular weight of 417 kDa was estimated for the enzyme present in a high-speed supernatant and was compared to 560 kDa calculated for the vacuolar α-mannosidase purified from *S. cerevisiae* [119]. The role of the soluble α-mannosidase of *C. albicans* in glycoprotein processing remained undetermined.

Further studies of α-mannosidases in *C. albicans* yeast cells resulted in the purification of two soluble forms of the enzyme, E-I and E-II, which were monomeric polypeptides of 54.3 and 93.3 kDa, respectively [119]. The enzymes were biochemically characterized using the fluorometric and colorimetric substrates described in [118]. Swainsonine and 1-deoxymannojirimycin strongly inhibited the hydrolysis of the fluorogenic substrate by both E-I and E-II, whereas the colorimetric substrate was slightly stimulated by E-I and not affected by E-II [119]. Linkage specificity of E-I and E-II was investigated with various substrates: CW mannans obtained from the wild type strain and the *mnn1* and *mnn2* mutants of *S. cerevisiae*, the AsnGlcNAc_2_Man_6_ (M_6_) oligosaccharide obtained from ovoalbumin, and the disaccharide 3-*O*-α-D-mannopyranosyl-D-mannopyranosyl (Man-α-1,3-Man). Released mannose from these substrates was analyzed by high-performance anion-exchange chromatography (HPAE) in Dionex equipment, Mod. LC20 Chromatography Enclosure. The results revealed that E-I and E-II from *C. albicans* preferentially cleaved α1,6 and α1,3 linkages, respectively [120].

A clue on the role of these enzymes in glycoprotein processing in *C. albicans* was obtained when E-I and E-II converted the yeast Man_10_GlcNAc (M_10_) oligosaccharide into an acceptor substrate of further mannose residues. Radiolabeled GDP-Man was used as a mannose donor, and the yeast MMF was used as the source of mannosyltransferases [121]. Accordingly, the M_10_ oligosaccharide was isolated from a triple mutant (*mnn1 mnn9 ldb2*) of *S. cerevisiae*. M_10_ lacks the characteristic signal phosphorylated molecule and contains a terminal α1,6-mannose substituted by an α1,2-mannose, making this product inert for further processing. In the first experiment, M_10_ was incubated at 37 °C with a buffer, E-I, or E-II. After 18 h, the released mannose was quantified by high-performance anion exchange chromatography. The results indicated that E-I and E-II released 1.2 and 2.0 mol of mannose per mol of M_10_, respectively. To obtain further insight into this activity, the radiolabeled sugar donor GDP-Man and other ingredients of the reaction mixture were incubated with either a buffer alone (A), MMF (B), MMF and M_10_ pre-incubated with a buffer (C), MMF and fresh M_10_ (D), MMF with M_10_ pre-incubated for 18 h at 37 °C with E-I (E), or E-II (F). Products formed after 18 h of incubation at 28 °C were separated in a column of Bio-Gel P-6, and the radioactivity of fractions was counted by liquid scintillation. Four mannose-labeled peaks of different sizes and amounts of radioactivity were obtained. Accordingly, a small peak, peak I, appeared in the void volume in eluates of incubation mixtures B–F. The nature of this peak was not investigated. Peak IV, with the minimum size, was recovered in all eluates and corresponded to the labeled sugar donor. Peak III appeared in an intermediate position between peaks I and IV in eluates of mixtures C–F and was not evident in the incubation mixture lacking M_10_ (B). Peak II, with a larger size than peak III, was detected in eluates of mixture F only. The label associated with peak III with respect to the total recovered radioactivity (sum of four peaks) varied from 10.3 (C, 2159 cpm) and 7.8% (D) to 18.1% (E) and 29.4% (F). In other words, the radioactivity and size of M_10_ significantly increased when it was pre-digested with E-I and, to a greater extent, with E-II. This indicates that these enzymes correspond to *N*-glycan processing glycosidases with E-I being more specific than E-II, as it removes a single mannose residue. In fact, the pre-digestion of M_10_ with E-II gave rise to a product (peak II) larger than peak III [121]. 

A soluble α1,2-mannosidase with the ability to hydrolyze Man_9_GlcNAc_2_ (M_9_) and Man_8_GlcNAc_2_ (M_8_) oligosaccharides was purified from *C. albicans* and was proposed as a new member of GH family 47. The enzyme was purified to a single polypeptide of 43 kDa by a conventional method of protein isolation [104]. The enzyme converted M_9_ into M_8_ isomer B and mannose after 12 h of incubation at 37 °C. The extension of time of incubation to 24 h resulted in the formation of Man_7_GlcNAc_2_ (M_7_) and another product, possibly Man_6_GlcNAc_2_ (M_6_). M_7_ and M_6_ oligosaccharides were also detected when M_8_ was used as a substrate. The enzyme failed to demannosylate Man_6_GlcNAc_2_-Asn, Man_5_GlcNAc_2_-Asn, and α1,6-mannobiosides, thus confirming enzyme specificity. The catalytic properties of the enzyme purified in this study strongly resemble the ER-resident α1,2-mannosidase. Yet, its molecular weight and linkage specificity disagree with those previously reported in [120]. This discrepancy may be explained by the difference in the isolation method used in these studies. Some of these reports are compiled in a short review [122].

Regarding α-glucosidases, a soluble α-glucosidase II was partially purified from yeast cells of *C. albicans*, and some of its properties were investigated [123]. Electrophoretic analysis of the purified preparation revealed two major polypeptides of 36 and 47 kDa. The latter was responsible for catalytic activity as visualized in zymograms with MUαGlc. Linkage specificity was investigated using nigerose, maltose, isomaltose, kojibiose, and trehalose as substrates. The enzyme showed a clear preference for nigerose, an a1,3-linked glucose disaccharide, over the other substrates, whose hydrolyses were barely significant. Moreover, the enzyme also converted GlcMan_9_GlcNAc_2_ into Man_9_GlcNAc_2_ oligosaccharide in a time-dependent manner. These properties are consistent with an α-glucosidase II involved in *N*-glycan processing. Later, by using protease inhibitors during cell processing, it was demonstrated that α-glucosidase II of *C. albicans* is a dimer of 83 kDa consisting of 36 and 47 kDa polypeptides [124]. The biochemical properties of the 47 kDa were essentially similar to those previously reported [123], suggesting that this protein is an α-glucosidase II released by the proteolysis of an 83 kDa precursor. 

It is well-documented that protein glycosylation plays a critical role in the pathogenesis and immune recognition of *C. albicans*. To obtain further evidence of the relevance of this process, homolog genes to *CWH41*, *ROT2*, and *MNS1*, which encode ER α-glucosidase I, α-glucosidase II catalytic subunit, and α1,2-mannosidase in *S. cerevisiae*, respectively, were disrupted in *C. albicans*, and the corresponding null mutant phenotypes were analyzed in terms of *N*-glycosylation, CW integrity, and host–fungus interaction [111]. The cells of *Cacwh41*, *Carot2*, and *Camns1∆* mutants showed several CW defects, exhibited attenuated virulence in a murine model of systemic infection, and stimulated the cytokine profile by human peripheral blood mononuclear cells (PBMCs), confirming that the processing of oligosaccharides by these ER glycosidases is crucial for the formation of high-mannose *N*-glycans, host–fungus interaction, and virulence. 

Despite the advance in the study of α-glucosidases I and II in *C. albicans* and other organisms, almost nothing is known about their 3D structure, catalytic amino acids, and specific residues that participate in substrate interaction. A study carried out with a recombinant α-glucosidase obtained from the expression of the *C. albicans CWH41* gene in *Escherichia coli* demonstrated that the recombinant peptide was catalytically active despite lacking 419 amino acids from the N-terminal end. The biochemical analysis of the enzyme revealed that the presence of hydroxyl groups at carbons 3 and 6 and the orientation of a hydroxyl moiety at C-2 are important for glucose recognition. Moreover, cysteine rather than histidine residues is in the catalytic pocket of the recombinant polypeptide [125]. 

## 4. Future Directions

While much progress has been made in the knowledge of *Candida* and candidiasis, research on *Sporothrix* species has mainly focused on the pathogenicity and virulence, epidemiology, diagnosis, and treatment of sporotrichosis, a neglected mycosis of humans and animals acquired by the routes discussed in this review. Only three members (*S. brasiliensis*, *S. schenckii,* and *S. globosa*) out of the fifty-three species in the genus impact human and animal health. Most of the other species belong to the environmental core and are not pathogenic for humans. Sporotrichosis is an expanding disease that can eventually reach a zoonotic epidemic driven by *S. brasiliensis* in cats and humans and become a serious threat to public health. Molecular studies on the physiology of these thermodimorphic fungi were seriously limited until the middle of the last decade, when the genome sequencing of *S. schenckii* and *S. brasiliensis* was announced in 2014. Hopefully, the identification and isolation of genes and the generation and phenotypic analysis of specific mutants in terms of altered cell functions will lead to a better knowledge of potential targets of antifungal drugs and a consequent improvement of the prevention and/or control not only of sporotrichosis but also other mycoses. Moreover, these approaches will strengthen genetic glycoengineering, as glycosylation/deglycosylation can be used to modulate the efficiency of protein pharmaceuticals, the modification of glycoprotein antibodies by adding or changing the position of some sugars, changing the properties of recombinant proteins, etc. These achievements will impact the areas of biotechnology, biomedicine, and consequently, human health.
